# The efficacy of kinesio tape in patients with lateral elbow tendinopathy: A systematic review and meta-analysis of prospective randomized controlled trials

**DOI:** 10.1016/j.heliyon.2024.e25606

**Published:** 2024-02-04

**Authors:** Yinghao Li, Lu Mei, Shugela Rahat, Long Pang, Ran Li, Yan Xiong, Jian Li, Xin Tang

**Affiliations:** a Sports Medicine Center, West China Hospital, Sichuan University, China; b Department of Orthopedics and Orthopedic Research Institute, West China Hospital, Sichuan University, China; c West China School of Nursing, Sichuan University, China; d Department of Rehabilitation Medicine, West China Hospital, Sichuan University, China

**Keywords:** Kinesio tape, Lateral elbow tendinopathy, Meta-analysis, Pain

## Abstract

**Background:**

The efficacy of Kinesio tape (KT) in lateral elbow tendinopathy (LET) has been widely discussed, but controversy remains.

**Objectives:**

To perform a meta-analysis of randomized controlled trials (RCTs) in the literature to ascertain the efficacy of KT in LET.

**Design:**

Systematic review and meta-analysis.

**Method:**

Two independent reviewers carried out a literature search in accordance with the PRISMA (Preferred Reporting Items for Systematic Reviews and Meta-Analyses) guidelines. Any discrepancies were addressed by a third author. Included in the study were RCTs comparing KT to a control group in the context of LET. The quality of evidence was assessed with the 2.0 version of Cochrane Collaboration risk of bias tool. Evaluation centered on clinical outcomes, such as function scores and pain, with comparison made using the risk ratio for dichotomous variables and the mean difference for continuous variables. Statistical significance was considered for *P* values < 0.05.

**Results:**

Included in this review are 11 RCTs with 562 patients. Significant results were noted in favor of KT compared with control based on the visual analog scale score at movement (SMD = −1.17; *P* = 0.03); visual analog scale score at movement (SMD = −1.08; *P* < 0.00001); maximal grip strength (SMD = 0.69; *P* < 0.00001); pain pressure threshold (SMD = 1.14; *P* < 0.00001); Patient-Rated Tennis Elbow Evaluation Questionnaire score (SMD = −1.16; *P* = 0.02) and Disabilities of the Arm, Shoulder, and Hand questionnaire score (SMD = −1.19; *P* < 0.00001).

**Conclusion:**

The current evidence shows that KT can improve pain levels and the function of elbow joint in patients with LET, and this improvement is might be clinically significant. We assume that physiotherapists can consider trying the KT in LET patients. Future quality studies are needed to confirm the efficacy and explore the mechanism of KT.

## Highlights

• KT can be effective for reducing pain and improving function in patients with LET.
• This study demonstrated clinically significant improvements in both VAS and PRTEE scores following KT treatment.
• Future high-quality studies with rigorous protocols are still needed to confirm the efficacy of KT.

## 1 Introduction

Lateral elbow tendinopathy (LET), also known as tennis elbow, is the most common cause of elbow pain in adults between 40 and 60 years of age with an annual incidence of 1 %–3 % [1,2]. The main lesion in LET is at the origin of the extensor carpi radialis brevis (ECRB) and is thought to arise after micro trauma and repetitive injury [1]. Long term contraction and tension of the forearm extensor can cause degenerative changes on the tendon. Generally, symptoms will resolve within a year with some non-operative managements as the first line of treatment. However, among the numerous treatment measures, no reliable conservative method has been confirmed to be able to prevent the progression of LET [[2], [3], [4]].

Kinesio tape (KT) was first introduced in 1979 in Japan, and currently it is the most common elastic taping [5]. It is assumed that it applies appropriate tension along the tape and places the target muscle in a stretched position [6]. By lifting the skin, KT may achieve some physiological effects such as increasing the inter-tissue space, reducing swelling, improving blood and lymph circulation, and enhancing proprioception [7,8]. It has been reported that KT may be able to not only relieve pain but also enhance functional status in patients with several kinds of musculoskeletal disorders [9,10], such as shoulder impingement syndrome [11] and motor function impairment in children with cerebral palsy [12]. However, some studies reported conflicting conclusions [13,14]. In LET cases, controversies regarding the efficacy of KT persist. Some randomized controlled trials indicated that KT was effective for decreasing pain intensity, recovering grip strength, and improving functionality in patients with LET [[15], [16], [17]]; while other studies demonstrated that the application of KT produced no effects on muscle strength and KT exerted an effect similar to that of a placebo [18,19].

A meta-analysis conducted by Zhong et al. [20] concluded that KT was effective in relieving pain, restoring grip strength, and improving functionality in patients with LET. However, with only 5 randomized controlled trials (RCTs) included, the reliability of the conclusion is impaired. What is more, several new high quality studies have been published since its publication and a new meta-analysis may be able to provide a more reliable conclusion with these studies included [19,21,22].

Due to the conflicting and low-quality evidence, we performed this meta-analysis of RCTs to ascertain the effectiveness of KT in cases of LET. Our hypothesis was that KT would alleviate pain and enhance joint function among patients experiencing LET.

## 2 Methods and material

### 2.1 Strategy of search

Two reviewers conducted a meta-analysis by examining the PubMed, Cochrane Library, Web of Science, and EMBASE databases independently. In addition, the reference lists of all retrieved articles were reviewed for potentially eligible studies. The following key terms were utilized for the search on March 17, 2023: ((Tennis Elbow) OR (Elbow, Tennis) OR (Elbows, Tennis) OR (Tennis Elbows) OR (Lateral Epicondylitis) OR (Epicondylitis, Lateral) OR (Epicondylitis, Lateral Humeral) OR (Humeral Epicondylitis, Lateral) OR (Lateral Humeral Epicondylitis) OR (lateral elbow tendinopathy)) AND ((Athletic Tape) OR (Tape, Athletic) OR (Orthotic Tape) OR (Tape, Orthotic) OR (Kinesio Tape) OR (Kinesio Tapes) OR (Tape, Kinesio) OR (Tapes, Kinesio) OR (Kinesio tape)). A senior author examined disparities in the inclusion or exclusion of studies.

## 3 Eligibility criteria

The inclusion criteria for this study were as follows: (1) RCTs comparing KT and a comparator such as sham taping, physiotherapy or other conservative treatments for lateral elbow tendinopathy; (2) published in English; (3) published in a peer-reviewed journal; and (4) full text available. The exclusion criteria for this study include: (1) retrospective studies; (2) basic science studies; (3) reviews; or (4) having incomplete data and the missing data could not be obtained by any means. Following the screening of titles and abstracts based on these criteria, the full texts of potentially relevant studies were thoroughly examined.

### 3.1 Risk of bias

Two reviewers, working independently, collected the data. The Cochrane Collaboration risk of bias tool 2.0, which includes the bias from selection, measurements and reporting, was used to evaluate risk [23]. A study was deemed to be “low risk” when every single item was scored as low risk. The classification of a study as "high risk" was made if any item received a high-risk score. Otherwise, the studies were deemed to be “some concerns”. Articles assessed to be “high risk” were excluded. Cohen's kappa was adopted to measure the interrater reliability and a kappa value of 0.8 or more was interpreted as *almost perfect agreement* [24]. The disagreements were resolved by a third, experienced rater.

### 3.2 Data collection

Every study included underwent evaluation based on tape type, tape shape, clinical outcomes, and radiologic outcomes. The key findings were as follows: (1) visual analog scale (VAS) score; (2) Patient-Rated Tennis Elbow Evaluation Questionnaire (PRTEE) score; (3) maximal grip strength (MGS) and pain-free grip strength (PFGS); (4) Disabilities of the Arm, Shoulder, and Hand (DASH) questionnaire score; and (5) pain pressure threshold (PPT).

### 3.3 Statistical analysis

All statistical analyses were performed using Review Manager (RevMan for Macintosh version 5.3; The Cochrane Collaboration). A sensitivity analysis was conducted by systematically excluding one study at a time and recalculating pooled outcomes for the remaining studies. Continuous outcomes were computed and presented as the standard mean difference (SMD), while dichotomous outcomes were expressed as the risk ratio (RR). Heterogeneity among studies was measured using the *I*^2^ statistic, with values of 25 %, 50 %, and 75 % indicating low, medium, and high heterogeneity, respectively. The fixed-effects model was applied when *I*^2^ < 50 %; otherwise, the random-effects model was used. To further assess heterogeneity, the 95 % predictive interval (PI) was calculated using R software (version 4.2.2). Publication bias was evaluated through a funnel plot, with P values < 0.05 considered statistically significant. The mean difference of VAS, PRTEE and DASH from every included study were compared with the minimal clinically important difference (MCID) to determine the clinical significance of the improvement. The MCID values were defined in accordance with the literature. KT was considered clinically effective when the MD between groups exceeded the following MCIDs: 1.5 points for VAS, 15.8 points for DASH, and 11 points for PRTEE [25]. This study has been registered on PROSPERO.

## 4 Results

### 4.1 Literature search

**Fig. 1:**
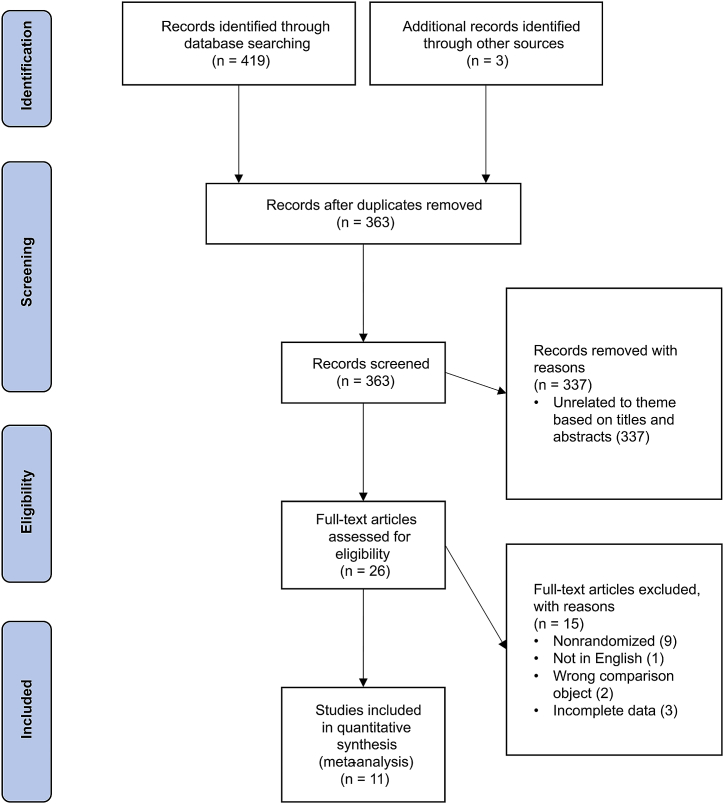
Preferred Reporting for Systematic Reviews and Meta-Analyses (PRISM) study selection flow diagram.

**Fig. 2:**
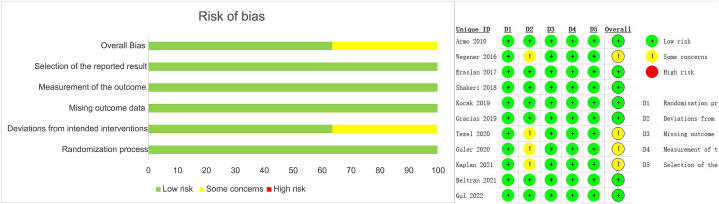
Results of the bias assessment of the included studies.

The search yielded a total of 422 studies. Following the elimination of 59 duplicate studies, the titles and abstracts of the remaining 363 articles were examined based on the previously mentioned eligibility criteria. Two researchers then examined the full texts of the 26 chosen studies. Included in this review were 11 RCTs [17,19,21,22,[26], [27], [28], [29], [30], [31], [32]] and the reporting bias was deemed to be low for all of them (Fig. 1). The Cohen's Kappa was 0.865, which indicated “Almost perfect agreement” [24]. The details of bias assessment are shown in Fig. 2.

### 4.2 Study characteristics and patient demographics

All included studies were level I or II prospective RCTs and the follow-up time ranged from 7 days to 6 months. In each study, the demographic characteristics of the 2 groups were similar. A total of 562 patients were included (285 in KT groups and 277 in control groups). The comparator groups received physiotherapy, sham tape or other conservative treatments while the KT groups received KT therapy on the basis of the comparator groups. Table 1 presents the characteristics of the study and the demographics of the patients.

**Table 1:** Characteristics of the included studies.

Author	Year	Follow-up	Application period of KT (w)	Sample size		Gender (M/F)		Age		Intervention of control
				KT	C	KT	C	KT	C	
Amro [26]	2010	4w	4	17	17	13/4	11/6	37.8	36.8	Traditional treatment
Wegener [32]	2016	3 m	12	14	13	3/11	5/8	47.38 ± 7.54	52.85 ± 7.14	Sham tape
Eraslan [17]	2017	6 m	3	15	15	NM	NM	48.5 ± 5.6	47.2 ± 8.5	Physiotherapy
Shakeri [30]	2018	1w	1	15	15	15/0	15/0	37.6 ± 11.56	31.62 ± 11.43	Sham tape
Kocak [29]	2019	12w	2.5	28	28	12/16	14/14	44.68 ± 9.83	43.54 ± 12.08	Steroid injection
Gracias [27]	2019	NM	NM	15	15	2/13	5/10	40 ± 6	35 ± 7	Sham tape
Tezel [31]	2020	3w	3	27	21	9/18	7/14	48.4 ± 10.6	46.8 ± 5.1	Sham tape
Guler [28]	2020	8w	3	20	20	6/14	7/13	44.8 ± 8.7	40.5 ± 7.9	ESWT
Kaplan [22]	2021	14w	14	40	40	14/26	14/26	45.1 ± 11.0	42.1 ± 7.0	Oral NSAIDs
Beltrán [19]	2021	1w	1	52	51	25/27	25/26	40.00 ± 8.22	41.08 ± 7.87	Sham tension
Gül [21]	2022	1 m	4	42	42	11/31	13/29	43.5 ± 8.3	46.0 ± 8.6	Traditional treatment

RCT, randomized controlled trial; KT, kenesio tape group; C, control group; w, week; m, month; d, day; NM, not mentioned; M, male; F, female; ESWT, extracorporeal shock wave therapy; NSAIDs, nonsteroidal anti-inflammatory drugs; SC, some concern.

### 4.3 Clinical outcomes

#### 4.3.1 VAS at rest

**Fig. 3:**
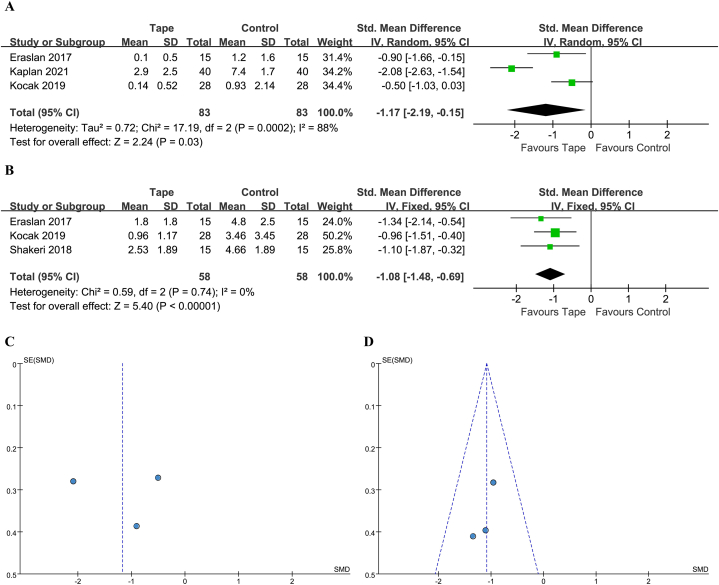
(A) Forest plot for VAS at rest. (B) Forest plot for VAS at movement. (C) Funnel plot for VAS at rest. (D) Funnel plot for VAS at movement. VAS, visual analog scale; CI, confidence interval; IV, inverse variance; Std, standard.

The VAS score at rest was reported in 3 studies [17,22,29], with 83 patients treated with KT and 83 with a control. One out of 3 studies reported a clinically significant difference. The heterogeneity was high and a random-effect model was used (*I*^2^ = 88 %, *P* = 0.0002). The 95 % PI was −4.01 to 0.76. A statistically significant benefit with KT was noted (SMD -1.17; 95 % CI -2.19 to −0.15; *P* = 0.03) (Fig. 3A&C).

#### 4.3.2 VAS at movement

The VAS score at movement was reported in 3 studies [17,29,30], with 58 patients treated with KT and 58 with a control. All three studies reported clinically significant differences. The heterogeneity was low and a fixed-effect model was used (*I*^2^ = 0 %, *P* = 0.74). The 95 % PI was −1.48 to −0.69. A statistically significant benefit with KT was noted (SMD -1.08; 95 % CI -1.48 to −0.69; *P* < 0.00001) (Fig. 3B&D).

#### 4.3.3 PFGS

**Fig. 4:**
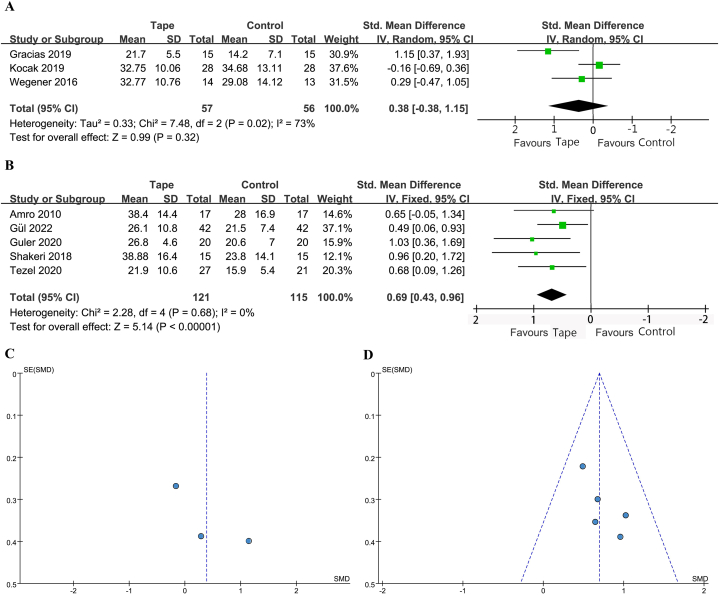
(A) Forest plot for PFGS. (B) Forest plot for MGS. (C) Funnel plot for PFGS. (D) Funnel plot for MGS. PFGS, pain-free grip strength; MGS, maximal grip strength; CI, confidence interval; IV, inverse variance; Std, standard.

The PFGS was reported in 3 studies [27,29,32], with 57 patients treated with KT and 56 with a control. The heterogeneity was high and a random-effect model was used (*I*^2^ = 73 %, *P* = 0.02). The 95 % PI was −1.92 to 4.24. No statistically significant difference was noted (SMD 0.38; 95 % CI -0.38 to 1.15; *P* = 0.32) (Fig. 4A&C).

#### 4.3.4 MGS

The MGS was reported in 5 studies [21,26,28,30,31], with 121 patients treated with KT and 115 with a control. The heterogeneity was low and a fixed-effect model was used (*I*^2^ = 0 %, *P* = 0.68). The 95 % PI was 0.43–0.96. A statistically significant benefit with KT was noted (SMD 0.69; 95 % CI 0.43 to 0.96; *P* < 0.00001) (Fig. 4B&D).

#### 4.3.5 PPT

**Fig. 5:**
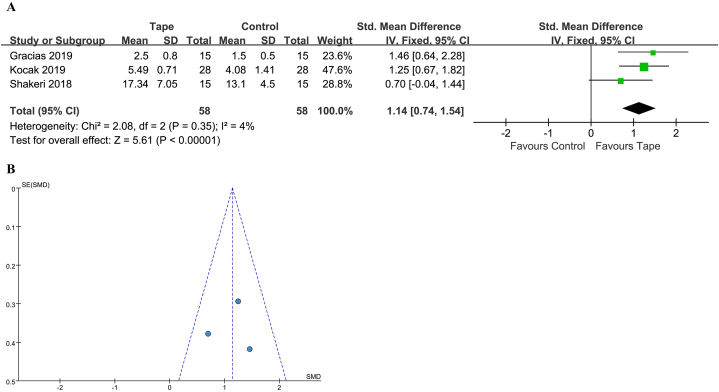
(A) Forest plot for PPT. (B) Funnel plot for PPT. PPT, pain pressure threshold; CI, confidence interval; IV, inverse variance; Std, standard.

The PPT was reported in 3 studies [27,29,30], with 58 patients treated with KT and 58 with a control. The heterogeneity was relatively low and a fixed-effect model was used (*I*^2^ = 4 %, *P* = 0.35). The 95 % PI was 0.74–1.54. A statistically significant benefit with KT was noted (SMD 1.14; 95 % CI 0.74 to 1.54; *P* < 0.00001) (Fig. 5A and B).

#### 4.3.6 PRTEE

**Fig. 6:**
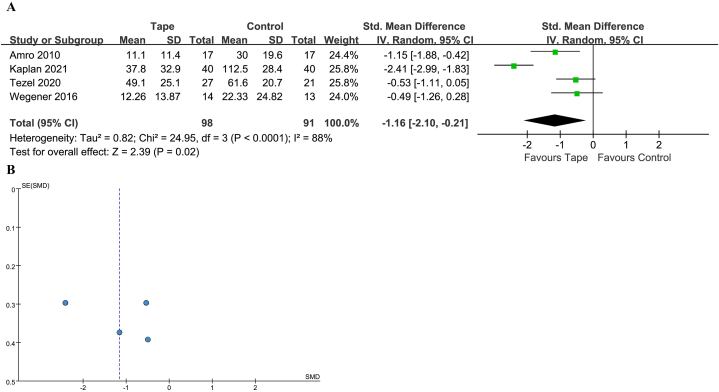
(A) Forest plot for PRTEE. (B) Funnel plot for PRTEE. PRTEE, Patient-Rated Tennis Elbow Evaluation Questionnaire; CI, confidence interval; IV, inverse variance; SD, standard deviation; Std, standard.

The PRTEE was reported in 4 studies [22,26,31,32], with 98 patients treated with KT and 91 with a control. None of the study reported clinically significant difference. The PRTEE score were measured differently in the study conducted by Kaplan et al. [22], so the SMD was adopted. The heterogeneity was significantly high and a random-effect model was used (*I*^2^ = 88 %, *P* < 0.0001). The 95 % PI was −15.68 to 3.96. A statistically significant benefit with KT was noted (SMD -1.16; 95 % CI -2.10 to −0.21; *P* = 0.02) (Fig. 6A and B).

#### 4.3.7 DASH score

**Fig. 7:**
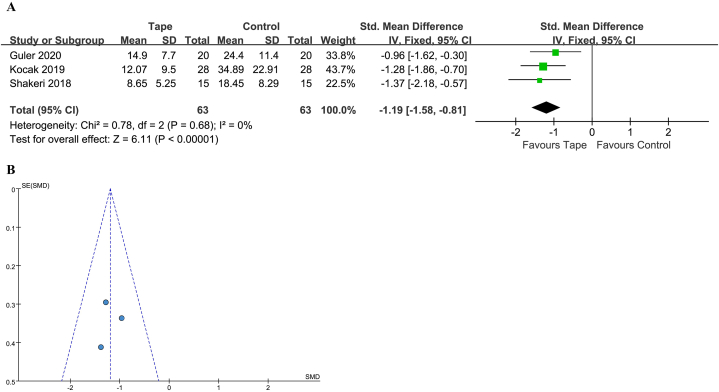
(A) Forest plot for DASH. (B) Funnel plot for DASH. DASH, Disabilities of the Arm, Shoulder, and Hand; CI, confidence interval; IV, inverse variance; Std, standard.

The DASH score was reported in 3 studies [[28], [29], [30]], with 63 patients treated with KT and 63 with a control. One out of 3 studies reported a clinically significant difference. The heterogeneity was low and a fixed-effect model was used (*I*^2^ = 0 %, *P* = 0.68). The 95 % PI was −1.58 to −0.81. A statistically significant benefit with KT was noted (SMD -1.19; 95 % CI -1.58 to −0.81; *P* < 0.00001) (Fig. 7A and B).

#### 4.3.8 Sensitivity analysis

The results of the sensitivity analysis concerning VAS at movement, PFGS, PPT and DASH score indicated that no significant effect was observed after removing any single study while those concerning VAS at rest and PRTEE presented different results. The *I*^2^ values were high in the analyses of VAS at rest, PFGS, and PRTEE, and the sources of the relatively high heterogeneity were found by conducting the sensitivity analysis. After the sensitivity analysis was performed, the results of PRTEE and VAS at rest changed and the *I*^2^ values in the analyses of VAS at rest, PFGS, and PRTEE decreased. The detailed information of sensitivity analysis is shown in Table 2.

**Table 2:** Results of SA

Outcome	Statistics before SA	Outcome Favors	Excluded Study	Statistics after SA	Outcome Favors
Changed results					
PRTEE	SMD -1.16; 95 % CI -2.10 to −0.21; *I*^2^ = 88 %; P = 0.02	KT	Amro 2010 [26]	SMD -1.15; 95 % CI -2.45 to 0.15; *I*^2^ = 92 %; P = 0.08	None
VAS at rest	SMD -1.17; 95 % CI -2.19 to −0.15; *I*^2^ = 88 %; P = 0.03	KT	Erasian 2017 [33]	SMD -1.29; 95 % CI -2.84 to 0.26; *I*^2^ = 94 %; P = 0.10	None
Reduced heterogeneity					
VAS at rest	SMD -1.17; 95 % CI -2.19 to −0.15; *I*^2^ = 88 %; P = 0.03	KT	Kaplan 2021 [22]	SMD -0.63; 95 % CI -1. 073 to −0.20; *I*^2^ = 0 %; P = 0.004	KT
PFGS	SMD 0.38; 95 % CI -0.38 to 1.15; *I*^2^ = 73 %; P = 0.32	None	Gracias 2019 [27]	SMD -0.02; 95 % CI -0.45 to 0.41; *I*^2^ = 0 %; P = 0.94	None
PRTEE	SMD -1.16; 95 % CI -2.10 to −0.21; *I*^2^ = 88 %; P = 0.02	KT	Kaplan 2021 [22]	MD -14.88; 95 % CI -22.17 to −7.59; *I*^2^ = 0 %; P < 0.0001	KT
SA, sensitivity analysis; PFGS, pain-free grip strength; PRTEE, Patient-Rated Tennis Elbow Evaluation Questionnaire; VAS, visual analog scale; SMD, standard mean difference; CI, confidence interval; KT, kenesio tape.					

## 5 Discussion

The results of the present study showed that the use of KT was associated with reduction in the VAS score at both rest and movement and improvement in MGS, PPT, PRTEE and DASH. These findings are partially consistent with our original hypothesis.

KT, a treatment commonly adopted in sports performance enhancement, injury prevention, and rehabilitation for occupational injuries [34], has gained popularity in recent decades and is currently widely used in musculoskeletal disorders [8,10]. This popularity may find support in the following studies. In an RCT, Dedeoglu et al. concluded that KT resulted in early control of pain and edema [35]. A meta-analysis performed by Li et al. argued that KT may improve the function and relieve the symptom of patients with hemiplegic shoulder pain [36]. Consequently, the efficacy of KT to relieve pain seems to make it a good candidate for the treatment of LET. However, controversy remains. Tezel et al. reported that both KT and sham taping provided similar improvement in pain relief [31]. Altas et al. confirmed that KT was effective on pain relief, functional capacity, and grip strength via ultrasound evaluation [37]. Behbahani et al. performed a systematic review to investigate the impact of KT in the treatment of LET, but the level of evidence was low since they included not RCTs but cohort studies and case-control studies [38].

LET is a clinical situation in which the patients’ intensity of pain, grip strength, and upper limb functionality were impaired [2]. These pivotal facets form the core dimensions of the assessment tools strategically integrated into the framework of this investigation. In this study, a noteworthy enhancement was observed across the majority of scores, barring the PFGS. This implies that the application of KT holds the potential to ameliorate both maximal strength and pain levels, subsequently fostering an enhancement in overall life quality while the intervention may not entirely eradicate symptoms of pain, particularly during activities demanding contraction of the ECRB.

Compared with the previous meta-analysis of RCTs conducted by Zhong et al. [20], we included more high-quality studies and excluded the studies conducted by Cho et al. [15] and Au et al. [39]. These studies are cross-over design studies, which compare the efficacy of the later method with that of the former. However, the LET has a nature of self-limit, which means the symptoms resolve spontaneously over time [40]. This would make the latter method seem to be more effective. What is more, we compared the inter-group differences from every study with MCID, which was not performed in previous studies but very important to make clinical decisions. The results demonstrated that clinically significant improvements in VAS at movement were detected in all the included studies. In terms of VAS at rest and PRTEE, the ratios were both 33.3 % (1/3). Considering that pain and restrict function are the main complains, this amelioration may bring more benefits for the patients with LET. Therefore, the affordable cost, broad applicability, and apparent lack of adverse reactions and contraindications, all together make KT a good option in treating LET.

Heterogeneity is a statistical indicator of homogeneity, as indicated by the *I*^2^ values, which were high in the analyses of VAS at rest, PFGS and PRTEE. A notable strength of this study lies in the adoption of a 95 % PI for analyzing heterogeneity, a novel approach not observed in prior research. The 95 % PI values elucidated that despite statistically significant differences in VAS at rest and PRTEE, the considerable heterogeneity necessitates cautious interpretation of these findings. Sensitivity analyses were conducted to pinpoint the origin of heterogeneity. The results proved that the studies conducted by Kocak et al. [29] and Kaplan et al. [22] were the main source of heterogeneity. The reason may be that in those two researches, patients in the control group received steroid injection or oral NSAIDs treatment, which can be a confounding factor.

This study has several limitations. Firstly, it exclusively incorporated RCTs published in English, potentially overlooking studies in other languages. However, this limitation is unlikely to introduce substantial bias, as the majority of high-quality studies are presently published in English. Second, the quality of this meta-analysis is restricted by the included studies. The relatively short follow-up duration, un-standardized confounding factors and small sample size of the original studies included restricted the strength of the conclusion in this study. Therefore, future high-quality studies are still needed. Last but not least, the heterogeneity is relatively high in the analyses performed. This can be explained since all the outcomes were continuous variables and according to a previous study, high statistical heterogeneity is more frequent in meta-analysis of continuous than binary outcomes [41]. In the meanwhile, sensitivity analysis was conducted and the relatively high heterogeneity is explicable.

## 6 Conclusion

The current evidence shows that KT can improve pain levels and the function of elbow joint in patients with LET, and this improvement might be clinically significant. We assume that physiotherapists can consider trying the KT in LET patients. Future quality studies are needed to confirm the efficacy and explore the mechanism of KT.

## Data availability statement

The data associated with our study has not been deposited into a publicly available repository because the data has been included in article/supplementary material/referenced in article.

## CRediT authorship contribution statement

**Yinghao Li:** Writing – review & editing, Writing – original draft, Methodology, Data curation. **Lu Mei:** Writing – review & editing, Validation, Data curation. **Shugela Rahat:** Software, Project administration, Methodology, Data curation. **Long Pang:** Visualization, Software. **Ran Li:** Software, Methodology, Formal analysis. **Yan Xiong:** Writing – review & editing, Validation. **Jian Li:** Writing – review & editing, Validation, Supervision. **Xin Tang:** Writing – review & editing, Validation, Supervision, Conceptualization.

## Declaration of competing interest

The authors declare that they have no known competing financial interests or personal relationships that could have appeared to influence the work reported in this paper.
