# Supplementary material for: The efficacy of kinesio tape in patients with lateral elbow tendinopathy: A systematic review and meta-analysis of prospective randomized controlled trials
Source: Heliyon. 2024 Feb 4;10(3):e25606. doi: 10.1016/j.heliyon.2024.e25606 (PMC10865320; doi:10.1016/j.heliyon.2024.e25606)
Supplement: Multimedia component 1 [file mmc1.docx]

Supplemental material 1: Searching strategy

Pubmed：

(Tennis Elbow[Title/Abstract]) OR (Elbow, Tennis[Title/Abstract]) OR (Elbows, Tennis[Title/Abstract]) OR (Tennis Elbows[Title/Abstract]) OR (Lateral Epicondylitis[Title/Abstract]) OR (Epicondylitis, Lateral[Title/Abstract]) OR (Epicondylitis, Lateral Humeral) OR (Humeral Epicondylitis, Lateral[Title/Abstract]) OR (Lateral Humeral Epicondylitis[Title/Abstract])

(Athletic Tape[Title/Abstract]) OR (Tape, Athletic[Title/Abstract]) OR (Orthotic Tape[Title/Abstract]) OR (Tape, Orthotic[Title/Abstract]) OR (Kinesio Tape[Title/Abstract]) OR (Kinesio Tapes[Title/Abstract]) OR (Tape, Kinesio[Title/Abstract]) OR (Tapes, Kinesio[Title/Abstract]) OR (Kinesiotape[Title/Abstract])

WOS:

TOPIC=(Tennis Elbow) OR TOPIC=(Elbow, Tennis) OR TOPIC=(Elbows, Tennis) OR TOPIC=(Tennis Elbows) OR TOPIC=(Lateral Epicondylitis) OR TOPIC=(Epicondylitides, Lateral) OR TOPIC=(Epicondylitis, Lateral) OR TOPIC=(Lateral Epicondylitides) OR TOPIC=(Epicondylitis, Lateral Humeral) OR TOPIC=(Epicondylitides, Lateral Humeral) OR TOPIC=(Humeral Epicondylitides, Lateral) OR TOPIC=(Humeral Epicondylitis, Lateral) OR TOPIC=(Lateral Humeral Epicondylitides) OR TOPIC=(Lateral Humeral Epicondylitis)

((lateral epicondylitis):ti,ab,kw OR (tennis elbow):ti,ab,kw) AND ((othotic Tape):ti,ab,kw OR (Kinesio Tape):ti,ab,kw)

TOPIC: (Athletic Tape) OR TOPIC: (Tape, Athletic) OR TOPIC: (Orthotic Tape) OR TOPIC: (Tape, Orthotic) OR TOPIC: (Kinesio Tape) OR TOPIC: (Kinesio Tapes) OR TOPIC: (Tape, Kinesio) OR TOPIC: (Tapes, Kinesio) OR TOPIC: (Kinesiotape)

'Tennis Elbow':ab,ti OR 'Elbow, Tennis':ab,ti OR 'Elbows, Tennis':ab,ti OR 'Tennis Elbows':ab,ti OR 'Lateral Epicondylitis':ab,ti OR 'Epicondylitides, Lateral':ab,ti OR 'Epicondylitis, Lateral':ab,ti OR'Lateral Epicondylitides':ab,ti OR 'Epicondylitis, Lateral Humeral':ab,ti OR 'Epicondylitides, Lateral Humeral':ab,ti OR'Humeral Epicondylitides, Lateral':ab,ti OR 'Humeral Epicondylitis, Lateral':ab,ti OR 'Lateral Humeral Epicondylitides':ab,ti OR 'Lateral Humeral Epicondylitis':ab,ti

('Tennis Elbow':ab,ti OR 'Lateral Epicondylitis':ab,ti) AND ('kenesio tape':ab,ti OR 'orthotic tape':ab,ti)

Pubmed:33

embase:213

CL:148

WOS:15

419 in total，

363 after duplication removal

Supplemental material 2: 95% predictive interval calculation

data <- data.frame(

exp_mean = c(X, X, X…),

exp_var = c(X, X, X…),

exp_sample = c(X, X, X…),

control_mean = c(X, X, X…),

control_var = c(X, X, X…),

control_sample = c(X, X, X…)

)

data$smd <- with(data, (exp_mean - control_mean) / sqrt((exp_var + control_var) / 2))

df <- data.frame(

effect = data$smd,

se = sqrt((data$exp_var / data$exp_sample) + (data$control_var / data$control_sample)),

group = rep(c("Experiment", "Control"), each = 3)

)

install.packages("metafor")

library(metafor)

meta_model <- rma(yi = effect, sei = se, data = df)

pi_result <- predict(meta_model, transf = exp, pi = TRUE)

print(pi_result)
